# Intact predictive processing in autistic adults: evidence from statistical learning

**DOI:** 10.1038/s41598-023-38708-3

**Published:** 2023-07-22

**Authors:** Orsolya Pesthy, Kinga Farkas, Laurie-Anne Sapey-Triomphe, Anna Guttengéber, Eszter Komoróczy, Karolina Janacsek, János M. Réthelyi, Dezső Németh

**Affiliations:** 1 Doctoral School of Psychology, ELTE Eötvös Loránd University, Budapest, Hungary; 2 Institute of Psychology, ELTE Eötvös Loránd University, Budapest, Hungary; 3 Research Centre for Natural Sciences, Institute of Cognitive Neuroscience and Psychology, Budapest, Hungary; 4 Department of Psychiatry and Psychotherapy, Semmelweis University, Budapest, Hungary; 5 Lyon Neuroscience Research Center (CRNL), INSERM U1028, CNRS UMR5292, Université Claude Bernard Lyon 1, Lyon, France; 6 Department of Clinical Psychology, Semmelweis University, Budapest, Hungary; 7 Faculty of Education, Health and Human Sciences, Centre for Thinking and Learning, School of Human Sciences, Institute for Lifecourse Development, University of Greenwich, London, UK; 8 BML-NAP Research Group, Institute of Psychology & Institute of Cognitive Neuroscience and Psychology, Eötvös Loránd University & Research Centre for Natural Sciences, Budapest, Hungary

**Keywords:** Human behaviour, Cognitive neuroscience

## Abstract

Impairment in predictive processes gained a lot of attention in recent years as an explanation for autistic symptoms. However, empirical evidence does not always underpin this framework. Thus, it is unclear what aspects of predictive processing are affected in autism spectrum disorder. In this study, we tested autistic adults on a task in which participants acquire probability-based regularities (that is, a statistical learning task). Twenty neurotypical and 22 autistic adults learned a probabilistic, temporally distributed regularity for about 40 min. Using frequentist and Bayesian methods, we found that autistic adults performed comparably to neurotypical adults, and the dynamics of learning did not differ between groups either. Thus, our study provides evidence for intact statistical learning in autistic adults. Furthermore, we discuss potential ways this result can extend the scope of the predictive processing framework, noting that atypical processing might not always mean a deficit in performance.

## Introduction

In the past years, several frameworks emerged to explain the neurocognitive mechanisms behind autism spectrum disorder (ASD). A line of research suggests that autistic behavior might emerge due to an atypical ability to predict future events based on experience and current sensory input—that is, predictive processing. The predictive processing framework originated from perception research: according to it, the brain generates hypotheses about the environment during perception based on previous experiences (priors) and updates the hypotheses using the prediction errors, that is, the differences between the predictions and the actual sensory inputs^1^. This framework has since been extended to a general framework for understanding brain functioning, including learning and memory^2,3^. It might benefit the understanding of ASD, and thus, help develop better supporting systems and interventions.

Various approaches to the predictive processing framework offer explanations for autistic traits by highlighting atypicalities in different components of the process. One of them assumes that autistic individuals tend to attribute a high and inflexible precision to prediction errors^3^. According to this view, autistic people would systematically adjust their internal representation of the world after each (minor) prediction error, instead of considering that some of these errors might simply signal unavoidable noise. Importantly, such errors indicate to the learner that the regularity is not fully learned yet^3,4^. Another viewpoint proposes that ASD individuals rely more on incoming sensory data (i.e., bottom-up information) compared to their prior experiences (i.e., top-down processes), which may result in less adaptive behaviour^4–8^. Lastly, in ASD, atypical predictive processing may arise from an inaccurate estimation of the extent to which environmental regularities change (as opposed to the estimation of the noise in the regularity itself, as mentioned above, see^9^ for different types of uncertainties), that is, the estimation of volatility^10^. Autistic people tend to overestimate volatility, even at the expense of learning environmental probabilities^8^. Altered or impaired predictive processes could explain sensory hypersensitivity^3,11^, deficits in sociocognitive skills^12^, and rigid habit-like behaviour in ASD (e.g.^3,4,10^). Despite its potential as a comprehensive framework for ASD, mixed empirical results on predictive processing suggest a more complex picture (for reviews, see^6,13^).

Predictive processing plays a crucial role in various functions, such as perception, different mechanisms of memory, and even habituation^2,14–16^. It can occur with^17^ or without^18,19^ reward. The former may be impaired or intact in ASD (e.g.^20^), depending on many factors, such as the reliability of the regularity to predict, whether the cues are social or nonsocial^21^, or the strength of the association^22^. However, the presence of reward or feedback can affect these results^23^, as reward sensitivity might be altered in ASD^24^. Thus, it is important to address examples of predictive processing that do not involve reward or trial-by-trial feedback.

Such is statistical learning: a form of predictive processing that entails learning probability-based regularities of the environment^25–27^. Despite its relevance, even the most comprehensive reviews often overlook or neglect studies about statistical learning in ASD^6,13^, although it contributes to language acquisition^28^, social skills^29^, and habit learning^30^—behaviors that are often altered in ASD^31^. Most statistical learning studies on ASD have used tasks where the regularity is predictable with a probability of one (that is, deterministic tasks). The results of these are mixed; some of them have found impaired^32–34^, and others have reported intact statistical learning in ASD^35–39^. Importantly, however, when regularities can be predicted with a probability less than one (often referred to as probabilistic regularities), no studies, to our knowledge, have found impaired statistical learning in ASD. Indeed, on probabilistic tasks, autistic individuals have similar^40,41^, or potentially even superior^42,43^ statistical learning performance compared to neurotypical peers. Thus, it appears that on probabilistic tasks, under certain circumstances, ASD participants perform similarly to (or even better than) neurotypical ones. What these circumstances are, however, is not fully understood.

Roser et al.^43^ used the differences in local and global processing to explain their results of superior statistical learning in autistic adults (compared to neurotypical adults). In their visuospatial task, Roser and colleagues presented participants with consecutive 3 × 3 grids containing abstract shapes. Unbeknownst to the participants, certain shapes consistently appeared in specific spatial relationships (e.g., two specific shapes always positioned diagonally to each other). The participants' (implicit) ability to differentiate these "base pairs" from other pairs was later assessed as a measure of their visual statistical learning. In this task, participants might benefit from local-level processing, which, importantly, would function superiorly in ASD (^44,45^; but see^46^ for contradicting evidence). Consequently, it is not clear whether superior performance in ASD measured by Roser et al.^43^ derived from better performance in statistical learning or just reflected differences in processing style. Thus, in our study, we aimed to test autistic individuals on a task where the performance assumably benefits less from local processing strategies, since regularities are temporally, and not spatially distributed^18^.

Note, however, that Roser et al.^43^ found superior learning only in autistic adults, but not in children (although they did not compare age groups directly). This is of importance, since statistical learning might change during the lifespan^19,47,48^. Although no study to date has compared the performance of autistic adults and children directly in a statistical learning task, the results of Roser et al.^43^ suggest that a superior statistical learning performance may only be present in autistic adults, but not in children, compared to their neurotypical age groups. Consequently, in our study, we aimed to compare the statistical learning performance of ASD versus neurotypical adults. We tested statistical learning using a probabilistic, temporally distributed task, where the pattern items do not follow each other directly but in a non-adjacent manner (the Alternating Serial Reaction Time (ASRT) task by Howard and Howard^18^). Based on Roser et al.^43^, we expected a superior performance of autistic compared to neurotypical adults.

## Methods

### Participants

In total, 45 participants were recruited for the study. Three neurotypical participants were excluded from the analysis due to errors in the data collection. Thus, the data of 42 participants were entered into the analyses, 20 of them were neurotypicals, and 22 of them had a diagnosis of ASD. Neurotypical participants were screened for diagnoses of any psychiatric or neurological disorders, and none of them scored higher on the autism spectrum quotient (AQ) questionnaire than 27, which means that they do not tend to show autistic behavioral patterns^49^. ASD diagnoses were provided by trained clinicians; both childhood scores of autism diagnostic interview-revised (ADI-R) and autism diagnostic observation schedule, IV-module (ADOS-IV)^50,51^ confirmed the diagnosis. We screened ASD participants for comorbid disorders: 12 of them had at least one of the following: attention deficit hyperactivity disorder (5), obsessive–compulsive disorder (3), generalized anxiety disorder (2), bipolar disorder (1), depression (1), and schizophrenia (1). Having an intellectual disability, language impairment, or active psychosis were exclusion criteria. Neurotypical participants were recruited by advertisement, while participants with ASD were recruited from the outpatient unit of the Department of Psychiatry and Psychotherapy, Semmelweis University. No participant received financial compensation for their participation.

The two groups did not differ in age, gender distribution, and years of education, see Table 1. All participants provided written informed consent. The study was conducted in accordance with the Declaration of Helsinki of 1975, as revised in 2008 and it was approved by the Regional and Institutional Committee of Science and Research Ethics, Semmelweis University, Budapest, Hungary (SERKEB No.: 145/2019). The experiment took place at the Laboratory of Brain, Memory and Language Lab, Eötvös Loránd University, Budapest.

**Table 1:** Demographic characteristics of the sample.

	Age (years)		Education (years)		Sex (f/m)		AQ		ADI-R (A + B + C)	ADOS (A + B)
	NTP	ASD	NTP	ASD	NTP	ASD	NTP	ASD	ASD	ASD
N					6/14	4/18				
Mean	25.40	27.32	16.00	15.98			15.20	31.09	36.68	9.95
SD	6.23	7.32	3.41	3.73			5.73	6.62	8.89	3.34
Minimum	19	19	12.0	9.5			5	15	20	5
Maximum	42	44	23.0	25.0			27	41	50	18
Statistics	*U* = 179.50, *p* = .312		*U* = 222.00, *p* = .970		*Χ*^2^ = 0.81, *p* = .369		*t(40)* = *− 8.28,* *p* < *.001*			

*AQ* autism-spectrum quotient, *NTP* neurotypical group, *ASD* autism spectrum disorder group, U = test statistics of the Mann–Whitney test, Χ^2^ = test statistics of the Chi-squared test. The sample size was N = 42. The ADI-R and ADOS scores apply only to the ASD group. Significant values are in [italics].

### Task and procedure

**Figure 1:**
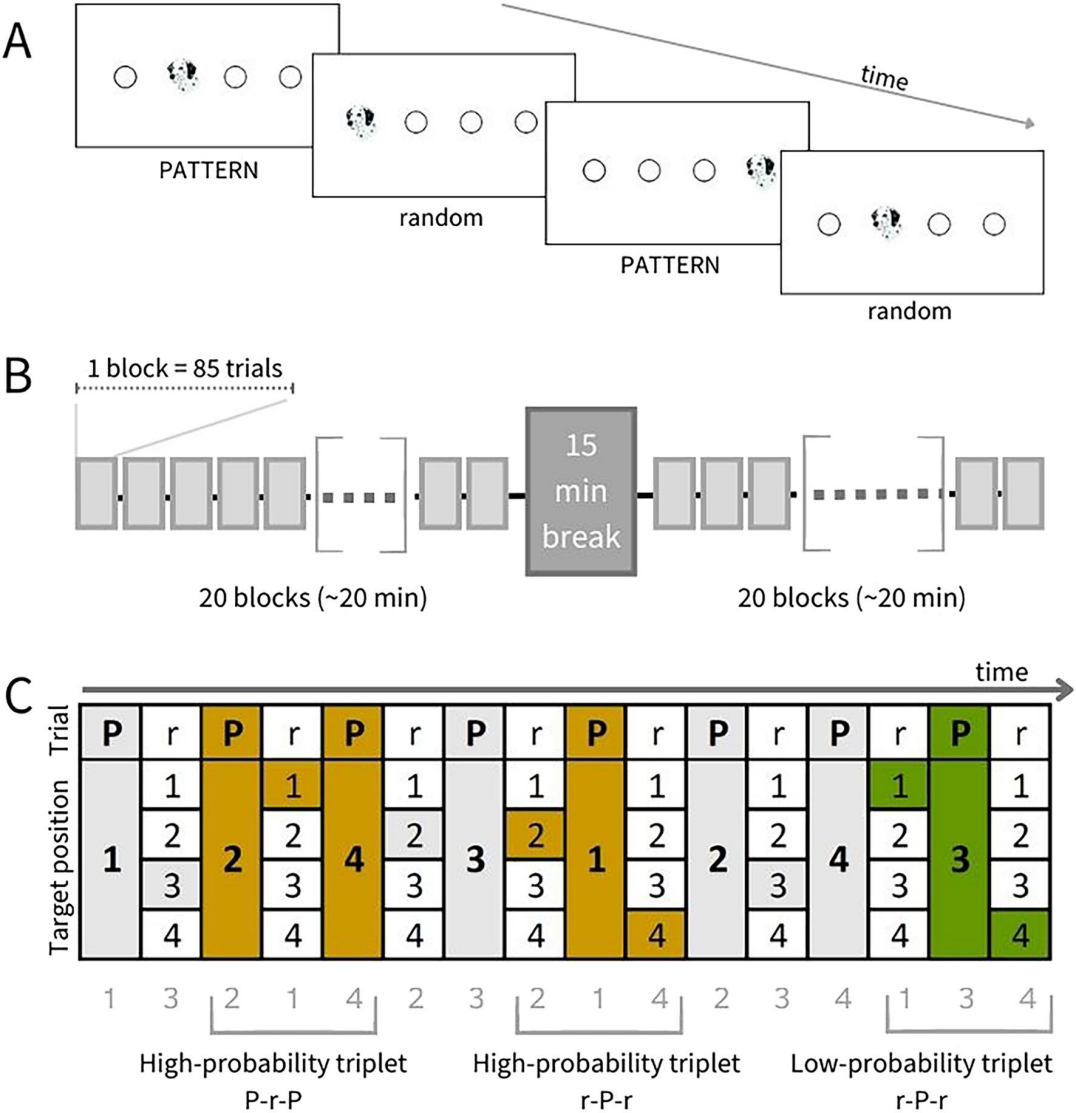
The task & design and an example sequence. (**A**) The grey rectangles represent the one-minute-long blocks. One block consisted of 85 trials and five blocks were merged into one unit of analysis (epoch). The stimulus appeared in one of the four locations. PATTERN and random stimuli alternated. (**B**) The design of the ASRT task. Participants performed for ~ 40 min in total, with a 15-min break in the middle. (**C**) Example for the sequence. High-probability triplets can be formed by two PATTERN (P) elements and one random (r), or by two random and one PATTERN element. Low-probability triplets can only be formed occasionally, by two random and one PATTERN elements; thus, they occur less frequently.

To measure statistical learning, we applied the ASRT task^18^, a commonly used and highly reliable task (e.g.^52^). In this task, participants saw four empty circles on a white background, horizontally arranged on the screen. A target stimulus (a dog’s head) appeared in one of the four locations. Participants were asked to press the button corresponding to the location of the appearing stimuli (Y, C, B, and M keys of a QWERTZ keyboard corresponded to the first, second, third, and fourth circle, from left to right respectively), using their right and left index and middle fingers. Participants were told that the goal of the task is to be as fast and as accurate as possible. Unknown to them, however, the serial order of the stimulus locations followed a specific structure: every second stimulus appeared randomly in one of the four possible locations, but every first element appeared systematically in the same order. Thus, these alternating elements formed an eight-element probabilistic sequence (e.g., 1r2r4r3r, where the numbers indicate the location of the elements belonging to the pattern, and r indicates a random position out of the four, see Fig. 1). Due to this structure, some combinations of three consecutive trials (triplets) were more likely to be formed. In the above example, 1 x 2, 2 x 4, 4 x 3, and 3 x 1 are high-probability triplets (where “x” indicates the middle element of the triplet, regardless of whether it is random or belongs to the pattern)—they can be both formed by two pattern and one random elements (PrP), or two random elements enclosing a pattern one (rPr). Out of the total of 64 possible triplets, 16 were high-probability triplets. Any other triplet (such as 1 x 3 or 2 x 1) cannot be formed by two pattern, and one random elements—thus, they occurred with low probability. Importantly, if participants perform with decreased RT and higher accuracy on the last element of a high-probability triplet (e.g., 2 in the above-mentioned 1 x 2 triplet) compared to the last element of a low-probability triplet (e.g., 3 in the above-mentioned 1 x 3 triplet), it means that the participant learned to predict the former one based on the preceding two elements, thus, acquired the underlying probability structure of the task. There were 48 low-probability triplets in this task. This task structure resulted in the following statistical structure: 50% of the trials were the last trial of a high-probability triplet formed by two pattern elements and one random (pattern-random-pattern), 12.5% of all trials were the last elements of a random-ending high-probability triplet (random-pattern-random). Therefore, high-probability triplets occurred with 62.5%, while low-probability triplets occurred with 37.5% overall probability. On the unique triplet level, high-probability triplets occurred with a 4% probability (62.5%/16), while low-probability ones occurred with a 0.8% probability (37.5%/48). As the last element of a high-probability triplet was more predictable than a low-probability triplet, we defined statistical learning as the difference in reaction times (RT) and accuracy performance between these triplet types. For further details of the ASRT task structure, see Fig. 1.

The task was divided into 40 blocks in total. Each block contained 85 trials: five random elements at the beginning (these were excluded from the analysis later), and an eight-elements alternating sequence ten times, as described above. The task was self-paced: the target stimulus remained on the screen until the first correct response, and the response-stimulus interval (RSI) was 120 ms, during which participants saw the four empty circles. Between blocks, participants received feedback on their RT and accuracy and could rest awhile. To reduce noise due to intra-individual variability in the analysis, we merged five blocks into one unit of analysis called an epoch.

To familiarize the participants with the ASRT task and to make sure they understood the instructions, participants first performed two blocks without the pattern (that is, all trials were random). After that, participants were asked to perform 8 epochs, with a ~ 15-min-long break after the 4th epoch. Despite the ASRT task being shown to be truly implicit (that is, no conscious knowledge is formed regarding the regularities hidden in the task, see^53^), once the ASRT was over, we administered a short questionnaire to make sure that none of the participants gained explicit knowledge of the structure of the task. It consisted of two questions increasingly specific to the nature of the structure: “Have you noticed anything special regarding the task?”, and “Have you noticed some regularity in the sequence of stimuli?”. According to this questionnaire, none of our participants gained conscious knowledge of the regularity.

### Statistical analysis

Statistical analyses were carried out using JASP 0.16.1.0^54^, and data preparation and visualization were conducted using Python 3.8, using pandas, NumPy, os, matplotlib, and seaborn packages ^55–57^. First, we determined about each trial in a sliding window manner whether, based on the two elements preceding it, they were the last element of a high- or a low-probability triplet (for the sake of simplicity, henceforth referred to as high-probability and low-probability triplets). That is, considering the example in Fig. 1, if the stimuli followed the “13214232” order, first, trial “2” was categorized as a high-probability triplet (1 3 2) element. Then, trial “1” was categorized as a high-probability triplet (3–2–1) element again, and so on. After this categorization, we excluded the last elements of trill (e.g., 2 1 2), and repetition (e.g., 2 2 2) triplets since participants show a pre-existing tendency to react faster to these elements, thus, they can bias the RTs^58^. We also screened for outlier trials using a boxplot, meaning that we excluded all trials where the RT fell outside the range of 1.5 inter-quartile distance (IQD) from the first quartile and 1.5 IQD from the third quartile. With this method, we excluded 5.83% of all trials in the entire sample (5.46% in the neurotypical, and 6.17% in the ASD group). Using the remaining data, we calculated the mean accuracy and median RT in each epoch, separately for high- and low-probability triplets. On these data, we performed a mixed-design ANOVA described in the Results section. When applicable, pairwise comparisons were performed using Holm correction.

Additionally to the frequentist statistics, we performed Bayesian analyses using default JASP priors, to be able to detect null results. Based on the BF_01_ values (which indicate the ratio of the likelihood of the null hypothesis to the likelihood to the alternative hypothesis), we calculated Bayes Factor_exclusion_ (BF_excl_) values. We compared the models to the null model (which included the subject variable and random slopes) in each case, and we calculated BF_excl_ values across matched models. BF_excl_ values indicate the likeliness of a model that does not include the given effect as opposed to the one that does. The BF_excl_ values above one rather support the exclusion of the given factor from the model, while values below one support the inclusion^59^. Values close to one mean that there is not enough evidence to support either inclusion or exclusion. We suggest a similar interpretation of these values as that of BF_01_ scores: a score above three means substantial evidence in favour of the null hypothesis, while a score between 0.33 and 1 indicates anecdotal evidence, while a score below 0.33 substantial evidence in favour of the alternative hypothesis^60,61^. For the sake of transparency, however, we reported BF_01_ values and errors (%) in Supplementary Materials S1 Table.

The data are available at https://osf.io/mebcx/.

### Significance statement

According to the predictive processing framework, autistic symptoms are the result of the weak ability to predict future events based on prior knowledge and sensory input. Despite its popularity, the validity of this framework and its limitations are still unclear. Here, we aim to test the predictive processing framework in autism by using a temporal statistical learning task. We found intact predictive processing in autism—neither the amount of learning nor the dynamics of it were altered. Our result challenges the predictive processing framework of autism. However, we suggest an update of the framework to better explain existing data and deepen our understanding of autism.

## Results

To test whether statistical learning differs between ASD and neurotypical groups, we conducted two mixed-design analyses of variances (ANOVAs), separately for accuracy and RT as dependent variables. In each, epoch (1–8) and triplet type (high/low-probability) served as within-subject factors and group (ASD/neurotypical) as a between-subject factor.

### Is statistical learning different between ASD and neurotypical adults? RT

We found a significant Triplet main effect in the ANOVA on RT [*F*(1,40) = 116.287, *p* < 0.001, *η*^*2*^_*p*_ = 0.744, *BF*_*excl*_ < 0.001]: participants were faster on the high- compared to the low-probability triplets, indicating that statistical learning was present throughout the task. According to the significant Epoch × Triplet interaction [*F*(7,280) = 7.162, *p* < 0.001, *η*^*2*^_*p*_ = 0.152, *BF*_*excl*_ < 0.001], this difference showed a gradual progress; reaching a significant level in the second epoch and remaining significant in every later epoch (*p*_Holm_ ≤ 0.021). Importantly, however, based on nonsignificant Triplet × Group and Epoch × Triplet × Group interactions, the groups differed neither in the overall amount of learning [*F*(1,40) = 1.603, *p* = 0.213, *η*^*2*^_*p*_ = 0.039, *BF*_*excl*_ = 2.828] nor in the dynamics of learning [*F*(7,280) = 0.720, *p* = 0.655, *η*^*2*^_*p*_ = 0.018, *BF*_*excl*_ = 25.586], respectively.

### Is statistical learning different between ASD and neurotypical adults? Accuracy

**Figure 2:**
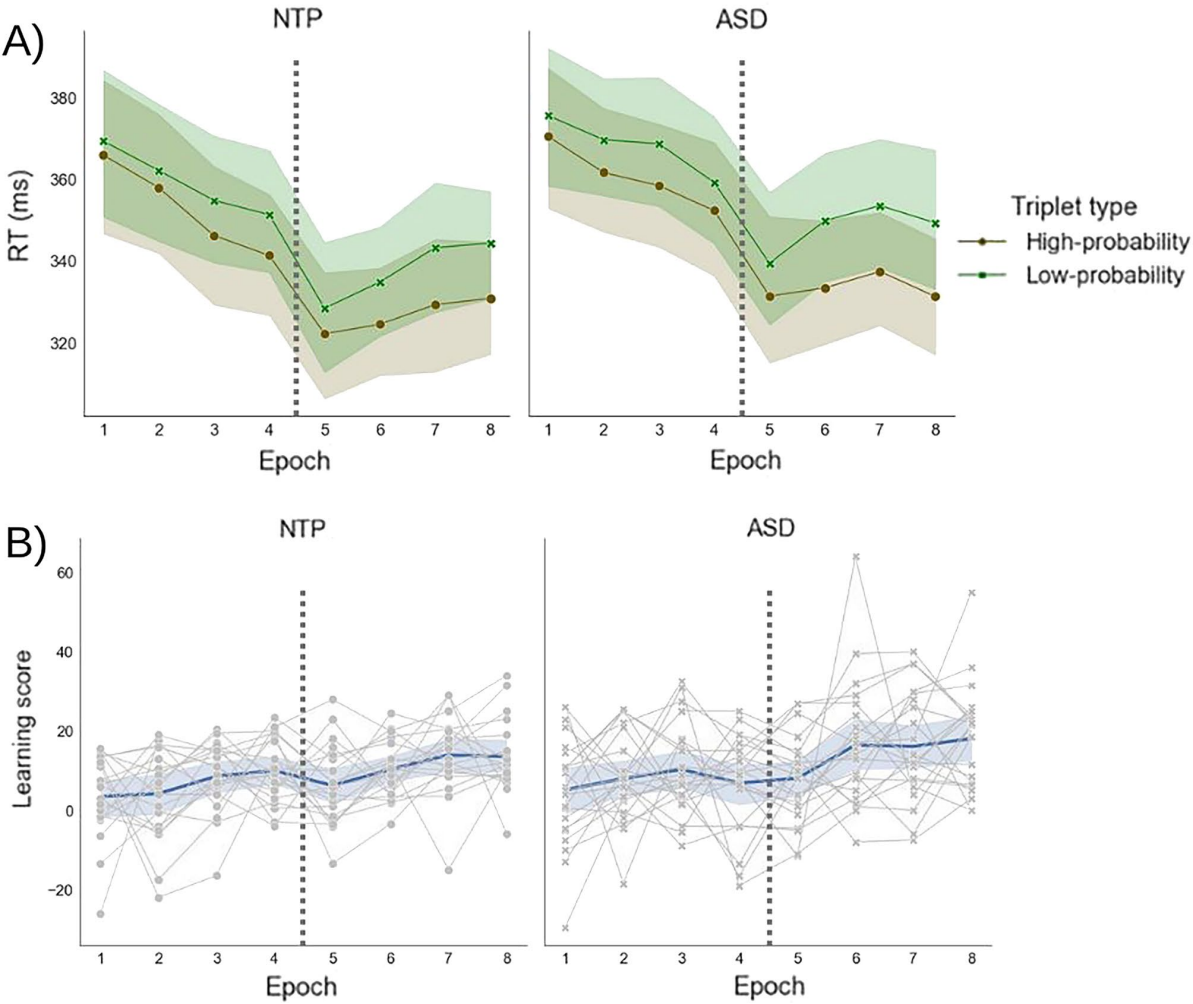
(**A**) Reaction time in the neurotypical (NTP, left figure) and ASD (right figure) groups, by the epochs. The brown color indicates the RT of high-probability triplets, and the green color the RT of low-probability triplets. The gap between these two lines indicates the magnitude of statistical learning. We found no significant differences between the groups. The dashed line indicates a 15-min long break. Error bands indicate the SEM. (**B**) Statistical learning score on RT, in the neurotypical (left figure) and ASD (right figure) groups, by the epochs. Learning scores indicate the RT differences between high- and low-probability triplets, i.e., show how many ms faster participants reacted to the high-probability vs. the low-probability triplets. The blue lines indicate the mean performance of the given group, and the gray lines represent the learning score of individual participants. The dashed line indicates a 15-min long break. We found no significant differences between the groups. Error bands indicate the standard error of the mean in the group.

The ANOVA on accuracy showed a similar pattern: the significant Triplet main effect indicated that statistical learning happened [*F*(1,40) = 33.805*, p* < 0.001*, η*^*2*^_*p*_ = 0.458, *BF*_*excl*_ < 0.01], i.e., participants were more accurate on the high- compared to the low-probability triplets. Moreover, there was a significant Epoch x Triplet interaction, showing a difference between epochs in the amount of learning, which, on the other hand, was not supported by the Bayesian statistics [*F*(7,280) = 2.443,* p* = 0.019, *η*^*2*^_*p*_ = 0.058, *BF*_*excl*_ = 1.333]—the difference between high- and low-probability triplets reached and maintained a significant level from the 4th epoch on (from that epoch on, *p*_Holm_ ≤ 0.003). Yet, both the overall learning [indicated by the Triplet × Group interaction: *F*(1,40) = 0.130, *p* = 0.721, *η*^*2*^_*p*_ = 0.003, *BF*_*excl*_ = 3.606] and the dynamics of learning [indicated by the Epoch × Triplet × Group interaction: *F*(7,280) = 0.898, *p* = 0.508, *η*^*2*^_*p*_ = 0.022, *BF*_*excl*_ = 15.263] were similar in ASD and neurotypical groups, see Fig. 2. Accuracy results are shown in Supplementary Materials (SM) Results Fig. S2. Results about the general, statistical learning-independent accuracy and RT are shown on Supplementary Fig. S3.

## Discussion

In this study, we aimed to test the statistical learning of autistic adults in light of the predictive processing framework. Besides the overall statistical learning, we also tested the dynamics of the learning process—which, to our best knowledge, has not been addressed in autistic adults before. We also performed exploratory analyses to find individual differences regarding the autistic symptom severity, which are reported in the SM (see Supplementary Information 1 and Supplementary Figure S3). Our findings provide frequentist and some Bayesian evidence of intact learning performance and similar learning curves in ASD and neurotypical participants.

These results seemingly contradict both the predictive processing framework of ASD that suggests impaired statistical learning in ASD^2,13^ and empirical findings by Roser et al.^43^, who found superior statistical learning in ASD. On the other hand, they are in line with previous literature that found no impairment in probabilistic statistical learning tasks in autistic children^36,40–42^. These contradictions highlight the possibility that predictive processing in autism might depend on the task used and that some aspects of it may be intact in ASD, which has both theoretical and clinical importance. In the following paragraphs, we will discuss possible explanations for these inconsistencies. First, the general information processing style the task requires might play a role. Second, atypicalities in different components of predictive processing could provide an explanation. As mentioned in the Introduction, the predictive processing framework of ASD is not a monolithic concept but rather an umbrella term that includes different mechanisms that could explain autistic traits/symptoms—these mechanisms are not necessarily mutually exclusive, yet apply a different angle to interpret the results. We did not directly access these mechanisms in our study, moreover, all these approaches face challenges by contradicting empirical results^20,62–64^. Thus, future studies are warranted on them, yet they may still help us understand our results in the context of the predictive processing framework and provide future directions. Lastly, we will discuss the potential role of age in statistical learning.

Based on the work of Roser et al.^43^, we even expected a superior statistical learning performance in ASD, as compared to neurotypical adults but could not replicate their results. An important difference between their task and ours was that their visual statistical learning task presented the learnable regularities on the same slide (that is, it was spatially distributed), whereas in our ASRT task, the learnable regularities were distributed in time (that is, temporally distributed). This leads to an important difference that might explain the contradictory results: the local- versus global-level processing involved in these tasks. Roser and colleagues^43^ argued that their findings were attributed to the significant engagement of local processing, a cognitive style in which autistic individuals often excel compared to neurotypical peers (^44^, but again, see^46^ for contradicting evidence). It is likely that our task, in comparison with the spatially distributed one used by Roser et al.^43^, requires more global-level integration: if participants fail to integrate the elements that successively occur, their statistical learning might be weaker. Although we acknowledge that acquiring spatially distributed regularities requires global-level integration as well, autistic individuals seem to benefit from a relative predominance of local-level processing^45^. Thus, the difference between our and Roser and colleagues’^43^ results may not at all derive from statistical learning, but from the atypicality of local/global processing.

Besides the general information processing style, atypically high and inflexible precision of prediction errors in ASD^3^ could account for the benefit of probabilistic tasks compared to deterministic ones. Such errors lead autistic people to update the model after each error, rather than contributing the errors to the unavoidable imprecision of the prediction itself. This has an important implication regarding our probabilistic statistical learning task: the constant update of the model might be adaptive in a task where the regularity cannot be fully learned due to its probabilistic nature. Thus, the constant update based on the prediction errors might lead to a longer learning process—the learning curve of neurotypical participants might peak sooner, as they do not update their model after a certain point, attributing the prediction errors to the imprecision of the otherwise correct model. Meanwhile, ASD participants might keep updating, thus, learning (see also the work of Gazzaniga^65^ about frequency-maximizing and frequency-matching strategies). This idea highlights the possibility that autistic predictive processing might depend on the given task type. Yet, this topic needs further investigation as some empirical evidence does not even support the different weighting of prediction errors in ASD (see^39^), and studies have suggested that some statistical learning tasks are not error-driven^66,67^.

It also implies that task length might affect ASD participants differently than neurotypical participants. Namely, neurotypical participants might outperform ASD participants on shorter tasks, but given enough time, ASD participants can catch up, or maybe even exceed the performance of neurotypical ones. Empirical evidence indeed supports this idea. Autistic participants tend to differ from neurotypical ones only in early learning^68^. Although they draw on prior knowledge less than neurotypical individuals, their priors are dominated by longer-term statistics of preceding stimuli, rather than recent ones^69–71^. Perhaps as a consequence of the above, they can catch up^72^ or even outperform their neurotypical peers by the end of the task (^42^—note, however, that this difference was only trend-level). Given enough time to learn, the constant updating of the representations might be adaptive in statistical learning. Another potential explanation is that, according to meta-analytic evidence, the overall global/local processing is similar in the autistic and neurotypical groups, but autistic people need more time for global processing than neurotypical people^46^—which might influence learning processes that require global processing. The slower learning dynamics might be an important methodological consideration, as most SRT/ASRT studies where ASD participants performed well, used longer (> 15 min long) learning sessions^40–42^—and our study, with about 40 min of practice provided another example for this. Taken together, the predictive processing of the autistic brain might lead to intact (or if supported by local processing, even superior) performance in case of probabilistic regularities. However, future studies shall address this question to be able to draw firm conclusions.

Atypical use of prior knowledge (vs. using primarily mere sensory input) in ASD might be another way to explain the results. Although empirical evidence often does not support the view that autistic individuals apply weak priors (e.g.^62–64^ for review see^6,7^), this might help to understand our results. Performance on probabilistic tasks might benefit more from bottom-up than top-down processes: one has to rely on bottom-up processes, as prior knowledge cannot predict the next event with a 100% probability. Thus, performance on the ASRT task potentially benefits more from bottom-up processes^26^ while using priors might even hinder it. With a real-life example, learning the grammar of a foreign language can be harder if we are proficient in another language already: the regularities we learned before in another language can automatically come to our minds instead of the correct grammar. In conclusion, while attributing lower weight to priors might harm performance on some predictive processing tasks, complex probabilistic task performance can even benefit from it.

A growing body of literature aims to capture another type of uncertainty in the prediction process. According to Palmer et al.^10^ and Lawson et al.^8^, autistic people in fact struggle with the estimation of volatility, rather than the estimation of the noise inherently present even when the regularity remains the same. Overestimating volatility leads to an aberrant learning process, which adds to the interpretation of our current results: although the ASRT task operates with some uncertainty (as in it is probabilistic), it is not volatile at all, which might explain the intact performance. This issue could be deeper understood by adding volatility to the ASRT task, for example by switching between different sequences to learn (see for example^73,74^). Such a study would provide insight into how different types of uncertainties affect learning in ASD. Moreover, using computational models such as hierarchical Gaussian filter would enable us to track the learning of volatility individually, c.f. Lawson et al.^8^. Given that volatility appears to offer an excellent explanation for our results, it would be particularly worthwhile for future studies to explore this concept.

However, statistical learning studies only ever have found an impairment in autistic children, not in adults. Moreover, all the previous studies that used our task showed no statistical learning impairment in autistic children^41,42^, which is in line with our findings on adults. All the studies to date, however, compare autistic individuals to neurotypical peers—to our knowledge, no study to date compared the statistical learning performance of autistic children with autistic adults—even though it might be of relevance, as statistical learning tends to change over the lifespan: neurotypical children can outperform adults on probabilistic tasks^19,47^. Most empirical evidence, including this current paper, suggests similar statistical learning throughout the lifespan in autistic and neurotypical individuals. On the other hand, the nature of the task (e.g., probabilistic/deterministic) might affect this as well, as results found on the SRT task in neurotypical children show a different developmental curve than on the ASRT task^47,48,75^, moreover, several functions show an altered developmental curve in ASD (see^7^ for review)—thus, we need further empirical evidence that directly tests this question.

Taken together, our paper aimed to investigate statistical learning in autistic adults from the predictive processing point of view. Predicting probabilistic, temporally distributed regularities seems to be intact, but not superior in ASD. It raises the possibility that predictive processing in ASD, even if it is atypical, can result in intact performance. Importantly, atypicality might affect the performance differently in seemingly similar tasks—here, we discussed how certain factors may contribute to predictive processing in ASD. We would like to inspire future studies not to consider predictive processing as a monolithic concept—for example, the same mechanisms might impair the performance in a deterministic task but not in a complex, probabilistic one. Furthermore, it might be useful for clinicians too; we suggest using strength-based methods in therapy and education of ASD patients, e.g., using probabilistic methods or giving enough time. These suggestions might help understand more about autistic predictive processing, and to autistic individuals to reach their best competencies.

## Supplementary Information

Supplementary Information 1.

Supplementary Figure S2.

Supplementary Figure S3.

## Data Availability

The raw datasets and the analyzed data for the current study are available at the following link: https://osf.io/mebcx/. The code to preprocess the raw data is available on GitHub: https://github.com/OrsPesthy/ASDstatlearning.
