# Supplementary material for: Intact predictive processing in autistic adults: evidence from statistical learning
Source: Sci Rep. 2023 Jul 22;13:11873. doi: 10.1038/s41598-023-38708-3 (PMC10363128; doi:10.1038/s41598-023-38708-3)
Supplement: Supplementary file 1 — Supplementary Information 1. [file 41598_2023_38708_MOESM1_ESM.pdf]

Intact predictive processing in autistic adults – evidence from statistical learning.

Supplementary Materials

Orsolya Pesthy[1,2,3**], Kinga Farkas[4**], Laurie-Anne Sapey-Triomphe[5], Anna Guttengéber[2,6], Eszter Komoróczy[4], Karolina Janacsek[2,7], János M. Réthelyi[4], Dezső Németh[2,3,5*]

[1] *Doctoral School of Psychology, ELTE Eötvös Loránd University, Budapest, Hungary*

[2] *Institute of Psychology, ELTE Eötvös Loránd University, Budapest, Hungary*

[3] *BML-NAP Research Group, Institute of Psychology, Eötvös Loránd University & Institute of Cognitive Neuroscience and Psychology, Research Centre for Natural Sciences, Budapest, Hungary*

[4] *Department of Psychiatry and Psychotherapy, Semmelweis University, Budapest, Hungary*

[5] *Lyon Neuroscience Research Center (CRNL), INSERM U1028, CNRS UMR5292, Université Claude Bernard Lyon 1, Lyon, France*

[6] *Department of Clinical Psychology, Semmelweis University, Budapest, Hungary*

[7] *Centre for Thinking and Learning, Institute for Lifecourse Development, School of Human Sciences, Faculty of Education, Health and Human Sciences, University of Greenwich, London, United Kingdom*

** Corresponding author*

*** These authors contributed equally to this paper*

Supplementary Results

General skill learning

On RT, participants became significantly faster, regardless of the triplet type [F(3.88, 155.16) = 75.93, p < .001, η^2_p = 0.66, BF_{excl} < 0.001], but as neither the Group main effect nor the Epoch x Group interaction was significant, neither the total reaction time nor the speedup during the performance was not significantly different in the groups [F(1,40) = 0.576, p = .452, η^2_p = 0.014, BF_{excl} = 1.254; F(3.88, 155.16) = 1.23, p = .300, η^2_p = 0.03, BF_{excl} = 4.257]. On the accuracy data, the Epoch main effect was significant [F(5.22, 209.01) = 9.793, p < .001, η^2_p = 0.20, BF_{excl} < 0.001], indicating an overall decrease in accuracy, regardless of the triplet types. The Group main effect and the Epoch x Group interaction was nonsignificant, thus, this change in accuracy was similar in the groups [F(1,40) = 0.180, p = .673, η^2_p = 0.004, BF_{excl} =1.842; F(5.22, 209.01) = 0.71, p = 0.623, η^2_p = 0.02, BF_{excl} = 26.809, respectively].

Bayesian analyses

Table S1.

Bayesian analyses: model comparisons

RT

**Models**	**BF_{01}**	**error %**
Null model (incl. subject and random slopes)	1	
EPOCH + TRIPLET + EPOCH * TRIPLET	6.09×10^{-76}	2.57
EPOCH + TRIPLET + group + EPOCH * TRIPLET	7.64×10^{-76}	3.76
EPOCH + TRIPLET + group + EPOCH * TRIPLET + TRIPLET * group	2.16×10^{-75}	1.63
EPOCH + TRIPLET + group + EPOCH * TRIPLET + EPOCH * group	3.25×10^{-75}	5.12
EPOCH + TRIPLET + group + EPOCH * TRIPLET + EPOCH * group + TRIPLET * group	9.18×10^{-75}	3.10
EPOCH + TRIPLET + group + EPOCH * TRIPLET + EPOCH * group + TRIPLET * group + EPOCH * TRIPLET * group	2.35×10^{-73}	2.62
EPOCH + TRIPLET	1.83×10^{-70}	1.27
EPOCH + TRIPLET + group	2.37×10^{-70}	2.50
EPOCH + TRIPLET + group + TRIPLET * group	6.11×10^{-70}	4.41
EPOCH + TRIPLET + group + EPOCH * group	1.15×10^{-69}	1.68
EPOCH + TRIPLET + group + EPOCH * group + TRIPLET * group	3.15×10^{-69}	2.72
EPOCH	3.63×10^{-60}	0.84
EPOCH + group	4.70×10^{-60}	1.15
EPOCH + group + EPOCH * group	2.39×10^{-59}	1.21
TRIPLET	5.04×10^{-11}	1.21
TRIPLET + group	6.39×10^{-11}	2.06
TRIPLET + group + TRIPLET * group	1.79×10^{-10}	2.86
group	1.31	0.92

Accuracy

**Models**	**BF_{01}**	**error %**
Null model (incl. subject and random slopes)	1	
EPOCH + TRIPLET	2.72×10^{-13}	2.93
EPOCH + TRIPLET + EPOCH * TRIPLET	3.02×10^{-13}	3.46
EPOCH + TRIPLET + group	3.62×10^{-13}	25.42
EPOCH + TRIPLET + group + EPOCH * TRIPLET	9.70×10^{-13}	37.66
EPOCH + TRIPLET + group + EPOCH * TRIPLET + TRIPLET * group	1.51×10^{-12}	60.73
EPOCH + TRIPLET + group + TRIPLET * group	2.35×10^{-12}	32.17
EPOCH + TRIPLET + group + EPOCH * TRIPLET + EPOCH * group	6.55×10^{-12}	69.00

EPOCH + TRIPLET + group + EPOCH ✻ group	5.72×10^{-11}	44.13
EPOCH + TRIPLET + group + EPOCH ✻ TRIPLET + EPOCH ✻ group + TRIPLET ✻ group	1.66×10^{-10}	45.04
EPOCH + TRIPLET + group + EPOCH ✻ group + TRIPLET ✻ group	1.77×10^{-10}	38.18
EPOCH + TRIPLET + group + EPOCH ✻ TRIPLET + EPOCH ✻ group + TRIPLET ✻ group + EPOCH ✻ TRIPLET ✻ group	2.53×10^{-9}	70.34
EPOCH	5.34×10^{-9}	0.90
EPOCH + group	1.06×10^{-8}	23.42
EPOCH + group + EPOCH ✻ group	1.63×10^{-7}	51.92
TRIPLET	5.43×10^{-5}	1.30
TRIPLET + group	9.83×10^{-5}	16.96
TRIPLET + group + TRIPLET ✻ group	4.14×10^{-4}	24.51
group	2.32	14.11

Note. All models include subject, and random slopes for all repeated measures factors.

Accuracy figures

Figure S2. Accuracy in the neurotypical (NTP, left figure) and ASD (right figure) groups, by the epochs. The brown color indicates the accuracy on high-probability triplets, and the green color the accuracy on low-probability triplets. The gap between these two lines indicates the magnitude of statistical learning. We found no significant differences between the groups. The dashed line indicates a 15-minute long break. Error bands indicate the SEM.

Exploratory analyses: correlations between ASD symptom severity and statistical learning performance

To test whether symptom severity affects learning performance, we ran correlation analyses. To do this, we calculated statistical learning scores for each epoch: we calculated how much faster and more accurate participants were on high- compared to low-probability triplets. Then, we correlated these scores with the AQ in the whole sample using Spearman’s correlation. We, however, found no significant correlations, see Figure S3. Please note, though, that our sample size was not designed to detect correlations, and that these analyses are highly exploratory.

Figure S3. Correlations between autism symptom severity measures and statistical learning scores. AQ = Autism-Spectrum Quotient. A) correlation between AQ and learning scores calculated on RTs, B) correlation between AQ and learning scores calculated on accuracy.

Post-hoc power analysis

To test the sufficiency of our sample size, we conducted a post hoc power analysis, using G*Power 3.1.9.7 [1]. Effect size *f*s were determined based on either the η^2_p reported in the main article (in the case of interactions) or the means, standard deviations, and Ns (in the case of group main effects), using the baseline settings of G*Power. We considered power above 80% sufficient. The results of this analysis for the effects relevant to our hypotheses (and for general skill learning, c.f. Supplementary Results) are shown in Table S1. Please note that regarding our hypotheses, the Triplet*group and Epoch*triplet*group effects are relevant. These effects reached sufficient power regarding RT. In accuracy, the Epoch*triplet*group interaction (which indicates the group differences in learning dynamics) was sufficiently powered, while the Triplet*group effect (indicating the group differences in the overall amount of learning) was underpowered. However, the null result on this effect is unlikely to be due to the lack of power – the Bayesian analysis (described in the main article) yielded sufficient evidence in favor of the exclusion of this effect from the model.

Table S2.

Achieved power of the relevant effects

Effect	Achieved power	Power sufficiency
RT		
Triplet*group	99.99%	Sufficient
Epoch*triplet*group	100%	Sufficient
Group	13.96%	Underpowered
Epoch*group	100%	Sufficient
Accuracy		
Triplet*group	15.36%	Underpowered
Epoch*triplet*group	99.97%	Sufficient
Group	8.25%	Underpowered
Epoch*group	93.88%	Sufficient

Supplementary References

1. Faul, F., Erdfelder, E., Lang, A. G. & Buchner, A. G*Power 3: A flexible statistical power analysis program for the social, behavioral, and biomedical sciences. *Behav Res Methods* **39**, 175–191 (2007).
