# Supplementary figures and images for: Intact predictive processing in autistic adults: evidence from statistical learning
Source: Sci Rep. 2023 Jul 22;13:11873. doi: 10.1038/s41598-023-38708-3 (PMC10363128; doi:10.1038/s41598-023-38708-3)

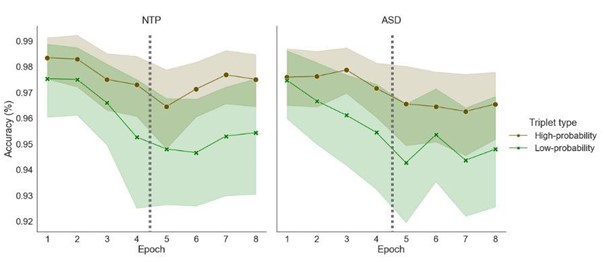

Supplement: Supplementary file 2 — Supplementary Figure S2. [file 41598_2023_38708_MOESM2_ESM.jpg]

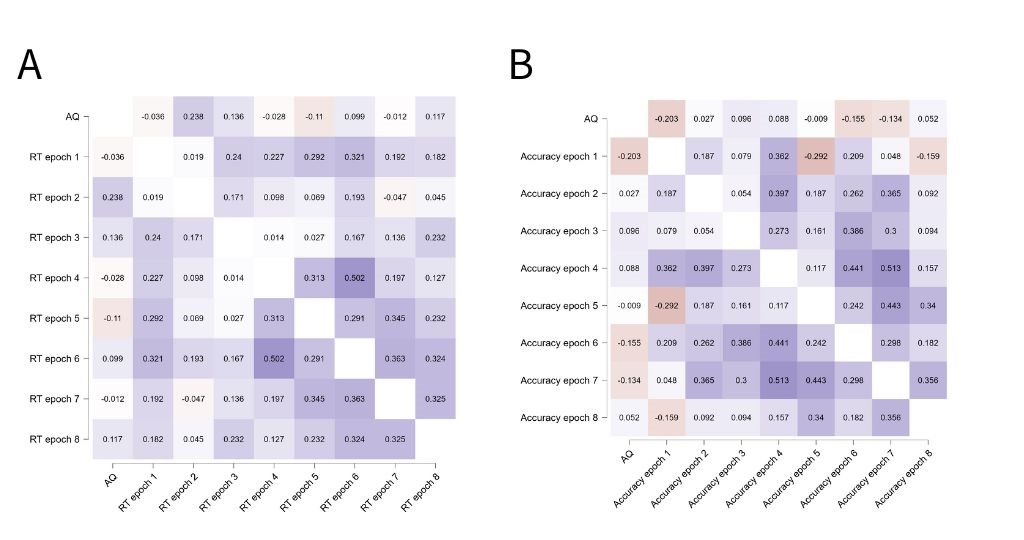

Supplement: Supplementary file 3 — Supplementary Figure S3. [file 41598_2023_38708_MOESM3_ESM.jpg]
